# A Metabolomic Profiling of Intra-Uterine Growth Restriction in Placenta and Cord Blood Points to an Impairment of Lipid and Energetic Metabolism

**DOI:** 10.3390/biomedicines10061411

**Published:** 2022-06-15

**Authors:** Juan Manuel Chao de la Barca, Floris Chabrun, Tiphaine Lefebvre, Ombeline Roche, Noémie Huetz, Odile Blanchet, Guillaume Legendre, Gilles Simard, Pascal Reynier, Géraldine Gascoin

**Affiliations:** 1 Unité Mixte de Recherche (UMR) MITOVASC, Structure Fédérative de Recherche (SFR) ICAT, Centre National de la Recherche Scientifique (CNRS), Institut National de la Santé et de la Recherche Médicale (INSERM), Université d’Angers, F-49000 Angers, France; floris.chabrun@chu-angers.fr (F.C.); pareynier@chu-angers.fr (P.R.); 2 Service de Biochimie et Biologie Moléculaire, Centre Hospitalier Universitaire, F-49000 Angers, France; gisimard@chu-angers.fr; 3 Réanimation et Médecine Néonatales, Centre Hospitalier Universitaire, F-49000 Angers, France; 85tiphaine.lefebvre@gmail.com (T.L.); rocheombeline@gmail.com (O.R.); noemiehuetz@yahoo.fr (N.H.); 4 Service de Médecine et Biologie de la Reproduction—Gynécologie Médicale, Centre Hospitalier Universitaire, F-44000 Nantes, France; 5 Centre de Ressources Biologiques, BB-0033-00038, Centre Hospitalier Universitaire, F-49000 Angers, France; odblanchet@chu-angers.fr; 6 Département de Gynécologie Obstétrique, Centre Hospitalier Universitaire, F-49000 Angers, France; guillaume.legendre@chu-angers.fr

**Keywords:** cord blood, intra-uterine growth restriction, metabolomics, lipidomic, placenta

## Abstract

(1) Background: Intrauterine growth restriction (IUGR) involves metabolic changes that may be responsible for an increased risk of metabolic and cardiovascular diseases in adulthood. Several metabolomic profiles have been reported in maternal blood and urine, amniotic fluid, cord blood and newborn urine, but the placenta has been poorly studied so far. (2) Methods: To decipher the origin of this metabolic reprogramming, we conducted a targeted metabolomics study replicated in two cohorts of placenta and one cohort of cord blood by measuring 188 metabolites by mass spectrometry. (3) Results: OPLS-DA multivariate analyses enabled clear discriminations between IUGR and controls, with good predictive capabilities and low overfitting in the two placental cohorts and in cord blood. A signature of 25 discriminating metabolites shared by both placental cohorts was identified. This signature points to sharp impairment of lipid and mitochondrial metabolism with an increased reliance on the creatine-phosphocreatine system by IUGR placentas. Increased placental insulin resistance and significant alteration of fatty acids oxidation, together with relatively higher phospholipase activity in IUGR placentas, were also highlighted. (4) Conclusions: Our results show a deep lipid and energetic remodeling in IUGR placentas that may have a lasting effect on the fetal metabolism.

## 1. Introduction

Intrauterine growth restriction (IUGR) or fetal growth restriction (FGR) generally refer to inadequate fetal growth with a birthweight below the 10th percentile according to gestational age and sex [1,2]. It affects up to 10% of pregnancies and increases the risk of perinatal morbidity and mortality, as well as the risk of long-term onset of metabolic and cardiovascular diseases in [3]. Prenatal identification of IUGR relies on ultrasound measurements, but this offers poor prognostic ability [1], and biomarkers of the disease are still lacking.

One of the branches of metabolomics, known as targeted metabolomics, consists in measuring absolute concentrations of a large but predefined set of metabolites. Metabolites are measured in samples from subjects with different clinical conditions (e.g., IUGR versus control placentas) and different statistical models are built to discriminate between affected and control samples. This hypothesis-free modeling of metabolomic data allows for the highlighting of candidate biomarkers, while shedding new light on pathophysiological mechanisms. Maternal blood, urine and hair, amniotic fluid, cord blood and newborn urine have been investigated using metabolomics in the context of IUGR [4,5]. These studies were recently subjected to a meta-analysis in which 15 publications were included [4]. Liquid chromatography paired with mass spectrometry was the most commonly used metabolomic approach, showing that fatty acids, phosphosphingolipids and amino acids were the most prevalent predictive metabolites. Vitamin D was the most prevalent predictive biomarker in the blood in the first trimester of pregnancy, the second one being homocysteine, an intermediate metabolite of DNA methylation, in the amniotic fluid in the second trimester. A deregulation of lipid metabolism, mostly fatty acids involved in energetic supply, was also observed in maternal blood.

In the blood of mothers, Sovio et al. reported a metabolomic signature predictive of FGR [6]. They performed untargeted metabolomics using UPLC-MS/MS in 175 FGR cases compared to 299 controls at 12, 20 and 28 weeks of gestational age, highlighting 22 discriminating metabolites. A ratio calculated from four of these metabolites showed good predictive performance with respect to FGR outcome (AUC = 0.78).

In neonates’ urine, increased levels of myo-inositol were found in FGR cases. Myo-inositol is known to regulate the free fatty acids released from adipose tissue and is potentially involved in metabolic syndrome [7,8].

Umbilical cord blood metabolome was shown to correlate strongly with birth weight, especially lysophosphatidylcholines, fatty acids and phosphatidylcholines, in the investigation of 700 cord blood samples (serum) by LC-MS/MS in a German cohort [9]. In cord blood, comparing IUGR (*n* = 22) and controls (*n* = 21) using LC-HRMS, Favretto et al. identified 22 discriminating metabolites, including phenylalanine, tryptophan and glutamate [10]. Comparing the cord blood serum of IUGR cases (*n* = 40) to controls (*n* = 40) using MS and NMR, Bahado-Singh et al. obtained an artificial intelligence-based discriminating model with an AUC = 0.91 [11]. Creatinine, acetyl carnitine (C2), butyl carnitine (C4), three lysophosphatidylcholines (lysoPC.a.C16.1, lysoPC.a.C20.3 and lysoPC.a.C28.1), and a phosphatidylcholine (PC.aa.C24.0) were the most discriminant metabolites in this model. The most altered metabolic pathways in IUGR were beta oxidation of very long fatty acids, oxidation of branched chain fatty acids, phospholipid biosynthesis, urea cycle, fatty acid and amino acid (lysine, tryptophan, cysteine and methionine) metabolisms. Interestingly, the comparison of cord blood plasma from early and late IUGR neonates showed distinct metabolomics profiles when NMR spectroscopy was used [12]. Glucose, acetone, glutamine and creatine concentrations were only affected in early IUGR (*n* = 23) compared to controls (*n* = 23), whereas valine and leucine levels were only affected in late IUGR (*n* = 56) compared to controls (*n* = 56), and both conditions showed an alteration of unsaturated lipids, VLDL, phenylalanine, tyrosine and choline concentrations.

Surprisingly, the placenta, the insufficiency of which is strongly involved in IUGR, has, to our knowledge, been the subject of only two recently published metabolomic studies [13,14]. The first one combined ^1^H NMR and LC-MS/MS study of the placenta in 19 cases of FGR, compared to 30 controls, identifying significant concentration differences in 179 metabolites [13]. This signature revealed that the main metabolic pathways affected were involved in the metabolism of bile acids, porphyrins, urea cycle, galactose and fructose, aspartate, tryptophan, proline and finally glycerophospholipids. The second study used ^1^H HR-MAS NMR to compare the placenta from 10 patients with FGR to 14 healthy controls [14]. This study showed increased concentrations of lactate, glutamine, glycerophosphocholine, taurine and myo-inositol in cases of FGR.

Here, we present a targeted metabolomics profiling of IUGR, conducted in cord blood and in two replicated placenta cohorts, and carried out using LC-MS/MS quantitative targeted metabolomics.

## 2. Materials and Methods

### 2.1. Patients

Placentas and cord blood samples were collected at the University Hospital of Angers, France. Maternal and fetal clinical data were collected from the patients’ obstetric records. All patients gave written consent for the use of their placenta. This study was authorized by the CPP (Comité de Protection des Personnes) ethics committee and registered with the French Ministry of Research under number DC-2011_1467. The cohort has also been registered with the CNIL (Commission Nationale de l’Informatique et des Libertés).

We included IUGR patients affected by poor placental perfusion (placental insufficiency), on the basis of clinical criteria with confirmed vascular anomalies seen on placentas, and excluded other causes of IUGR such as maternal hypertensive, metabolic and genetic diseases. Placentas were obtained from caesarean sections before or during the onset of labor, or from vaginal delivery. For the analysis, patients were classified into two groups, Intra-Uterine Growth Restriction (IUGR) and control. IUGR was defined by a reduction of fetal growth during gestation, with a notch observed by Echo-Doppler in at least one uterine artery and with Doppler abnormalities on umbilical Doppler and/or cerebral Doppler and/or ductus venosus, and with a birth weight below the 10th percentile according to the Audipog growth curve [1] and confirmed by the anatomopathological analysis of the placenta after birth. The control group was defined by women with normal pregnancies and who underwent a planned caesarean section before labor at term. Two cohorts of IUGR placental tissue, hereinafter named P1 (*n* = 20 IUGR versus 20 controls) and P2 (*n* = 24 IUGR versus 22 controls), were collected over a period of two years (2016–2017). Most of the women included in the IUGR group gave birth after a caesarean section (15/20 in P1 and 22/24 in P2) (Tables S1 and S2). These two cohorts differed only in their IUGR level of severity, the second cohort being more severe with a lower term age.

### 2.2. Placental and Cord Blood Samples

All placental tissues were dissected within 30 min after delivery. The protocol for placental dissection has been described previously [15]. Briefly, after removal of maternal decidua and amniotic membranes, 1 cm^3^ sections of placental villi were dissected from four different cotyledons between the basal and chorionic plates. After washing with PBS to remove maternal blood, the tissues were frozen at −80 °C until metabolites were extracted. Placentas were then sent for pathological analysis and stored at the biological core facility at Angers University Hospital (Centre de Ressources Biologiques).

Cord blood samples were collected from the umbilical vein. The blood transported in ice was immediately centrifuged for 15 min at 3000 rpm, and the supernatant (plasma) was stored as aliquots at −80 °C until metabolomic analysis. We included samples from both P1 and P2 cohorts for cord blood analysis: 15 in the IUGR group (4 P1 + 11 P2) versus 15 in the control group (8 P1 + 7 P2) (Table S3).

### 2.3. Metabolite Extraction and Protein Quantification from Placental Tissues

Placental tissues weighing between 20 and 50 mg were thawed on ice before being transferred to a 500 μL Precellys^®^ tube filled with ceramic beads. Forty microliters of cold water were added to the Precellys^®^ tube and tissues were grinded at 6500 rpm for 40 s. Five microliters of the supernatant obtained after centrifugation at 12,000× *g* for 5 min at 4 °C were taken for protein concentration determination. Two hundred microliters of supernatant were added to Precellys^®^ tubes and submitted to another grinding cycle of 6500 rpm for 40 s. After centrifugation at 16,000× *g* for 5 min at 4 °C, 100 μL of the supernatant were stored at −80 °C until mass spectrometry (MS) analysis. Protein concentrations were measured using a colorimetric method using bicinchoninic acid following the manufacturer’s instructions (BC Assay kit, Interchim, Montluçon, France).

### 2.4. Metabolomic Analysis Using Biocrates^®^ Technology

A targeted quantitative metabolomics approach was performed on placental and cord blood plasma extracts using the Biocrates AbsoluteIDQ p180 kit (Biocrates Life Sciences AG, Innsbruck, Austria) and an AB Sciex QTRAP 5500 mass spectrometer (SCIEX, Villebon sur Yvette, France). This kit allows the quantification of 188 metabolites, including 40 acylcarnitines, 21 amino acids, 21 biogenic amines, 90 glycerophospholipids, 15 sphingolipids and the sum of hexoses. Liquid chromatography (LC) was used to separate amino acids and biogenic amines before detection by tandem mass spectrometry (LC-MS/MS), whereas flow injection analysis with tandem mass spectrometry (FIA-MS/MS) was used to quantify acylcarnitines, glycerophospholipids, sphingolipids, and sugars. Ten μL of each sample (placenta homogenate supernatant or plasma) was added to the center of the filter placed on the top wall of the well in a 96-well plate. Metabolites were extracted in methanol solution using ammonium acetate after drying the filter spot under nitrogen flow and derivatization with phenylisothiocyanate for quantification of amino acids and biogenic amines. After validation of the three quality control levels, metabolite concentrations were used for statistical analyses only if they fell within the quantification range determined by the calibration curves. Metabolites with more than 20% of their values outside the range of quantification were not considered. Before excluding these metabolites, a χ2 test was performed to verify whether being outside the range of quantification was independent of the IUGR/control comparison.

### 2.5. Statistical Analyses

The Student’s *t*-test was used to compare the metabolite concentrations in placenta and newborn cord blood samples for the IUGR and control groups. The non-parametric Mann–Whitney–Wilcoxon test was used to compare metabolite sums and ratios between these cohorts. The Benjamini–Hochberg correction was applied to account for risk I inflation associated with multiple comparisons.

Metabolites were scaled to have zero mean and unit variance (UV scaling) before submission to unsupervised and supervised algorithms. Principal component analysis (PCA) and orthogonal projection to latent structures-discriminant analysis (OPLS-DA) were the unsupervised and supervised methods used in multivariate analysis. PCA enables outlier detection, based on Hotelling’s T2 distance, and identification of similar samples grouping together in the scatter plot. In the supervised analysis, the X matrix of predictive variables was composed of metabolite concentrations and the Y vector contained the information relative to the group (control or IUGR). To avoid selecting optimistic but over-fitted models, the predictive capabilities of the OPLS-DA models were evaluated by cross-validation using cross-validated R^2^Y (Q^2^Ycum or goodness of prediction), the cross-validated analysis of variance (CV-ANOVA) test, and the goodness of prediction of permuted models (Q^2^Y cum-perm). Models with a low degree of over-fitting are characterized by Q^2^Ycum > 0.5, negative Q^2^Y cum-perm and are significantly more discriminant than the null model (*p*-value _CV-ANOVA_ < 0.05). In predictive models, selection of metabolites of interest was made through the combination of two pieces of information: variable importance in the projection (VIP) and the loading between the metabolite in the X matrix and the predictive latent variable(s) of the OPLS-DA models. Only metabolites with a VIP value larger than 1 and absolute high loading values were considered as important in the metabolomics signature.

## 3. Results

### 3.1. Clinical Description

Placenta P1 and P2, and cord blood cohorts are described in Supplementary Tables S1–S3, respectively. The main difference in P1 and P2 is the term of birth of the IUGR group. Indeed, newborns in the IUGR group are more preterm in P2 than in P1 (31.3 vs. 36.4 weeks of gestational age, respectively). The term of birth in the control group is thus closer to that of the IUGR group from the P1 cohort. Nevertheless, IUGR was more severe in newborns from the P1 cohort than P2 (−2.2 vs. −1.8 mean birth weight z-score). All the women included in the control group gave birth after a planned caesarean section. Most of the women included in the IUGR group gave birth after a caesarean section (15/20 in P1 and 22/24 in P2).

### 3.2. Metabolomic Signature of P1 Cohort

**Figure 1:**
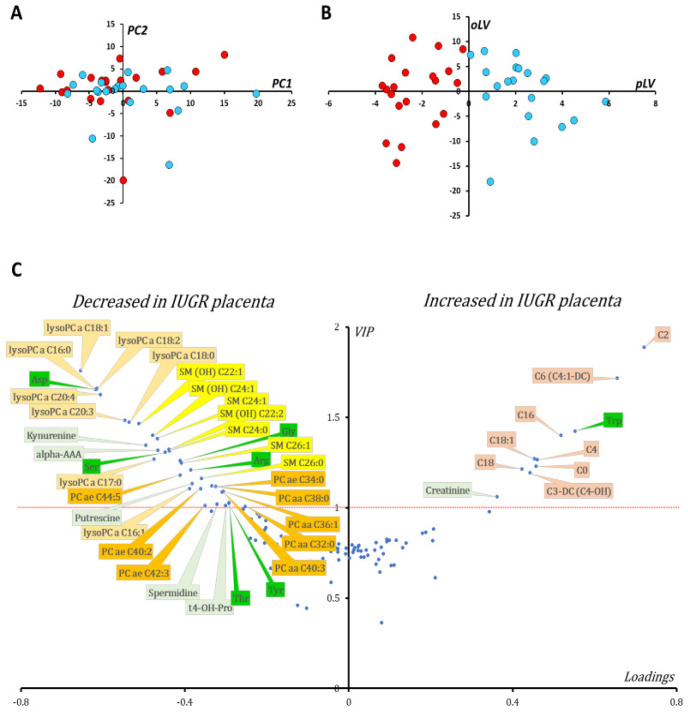
Multivariate statistical analysis of the metabolomic data from P1 cohort. Samples do not group together according to the IUGR status (red dots for IUGR and blue dost for controls) in the scatter plot of the PCA first principal plan (**A**), nor do any outliers appear according to Hotelling’s T2. When the OPLS-DA model is built, projecting the samples onto the predictive latent variable (pLV) enables perfect discrimination between IUGR and control samples (**B**). Loadings vs. VIPs (volcano plot, (**C**)) show increase concentrations of carnitine (C0) and long (C16, C18, C18:1) and short (C2, C6, and C4) acylcarnitine species along with tryptophan (Trp) and creatinine. On the other hand, many lipids, including lysophosphatidylcholine (lyso PCs), sphingomyelin (SM) and hydroxy sphingomyelin (SM(OH)) as well as some phosphatidylcholine (PC) species, are increased in the IUGR group compared to controls. The concentration of some polar metabolites is also decreased in placentas from the IUGR group, such as the amino acids aspartate (Asp), serine (Ser), glycine (Gly), arginine (Arg), threonine (Thr) and tyrosine (Tyr), as well as biogenic amines including kynurenine, alpha-aminoadipic acid (alpha-AAA), two polyamines (putrescine and spermidine) and the collagen-degradation product trans-4-hydroxyproline (t4-OH-Pro). For phosphatidylcholines, the sum of the length of the two acyl or acyl-alkyl groups is noted after the C and is followed by the number of double bonds. The same notation is used to represent the length and the number of double bonds in the acyl chain of sphingomyelins and lysophosphatidylcholine species. Color code: amino acids: green; carnitine and acylcarnitine species: brown; biogenic amines: light green; lysophosphatidylcholine species: light orange; phosphatidylcholine species: dark orange; sphingomyelins and hydroxy sphingomyelins: yellow.

From 188 measured metabolites, 127 were in the quantitation range and were kept for statistical analyses (see Supplementary Table S1). PCA showed no outliers nor spontaneous sample grouping (Figure 1A). The OPLS-DA method enabled good group discrimination (R^2^Y = 0.85) as observed in Figure 1B, with good predictive capabilities and a low risk of overfitting (Q^2^Y cum = 0.72; *p*-value _CV-ANOVA_ < 0.0001; Q^2^Y cum-perm = −0.61). Metabolite ranking according to VIP and loadings are presented in Figure 1C.

Multivariate supervised analysis showed increased concentration of carnitine and acylcarnitine species (AC) (short chain length C2, C3-DC, C4 and C6 and long chain length C16, C18 and C18:1) in IUGR placentas compared to controls. Furthermore, the ratio between acetyl and propionyl carnitines (C2 + C3) and free carnitine (C0), an indicator of fatty acid β-oxidation, was significantly increased in IUGR placentas (median fold change of 1.49, *p*-value _Wilcoxon_ = 0.006). Creatinine and tryptophan were also relatively increased in IUGR placentas. Kynurenine, a metabolite derived from tryptophan, was decreased in IUGR placentas, making the kynurenine/tryptophan ratio significantly diminished in IUGR compared to controls (median fold change of 0.13, *p*-value _Wilcoxon_ < 0.0001). Arginine was also diminished in IUGR placentas with relatively increased activity of arginase in this group, as measured by the ornithine/arginine ratio (median fold change of 1.39, *p*-value _Wilcoxon_ = 0.0002).

The concentration of other amino acids such as aspartate, glycine, serine, and threonine was also found to be relatively lower in placentas from the IUGR group compared to controls. The polyamines putrescine and spermidine were found to be diminished in IUGR placentas with increased activity of spermine synthase, quantified by the spermine/spermidine ratio (median fold change of 1.26, *p*-value _Wilcoxon_ = 0.001).

A deep lipid remodeling was also observed when comparing IUGR and control placenta. Lysophosphatidylcholine species of less than 22 carbon atoms were diminished in IUGR placentas compared to controls with average values of 4.97 and 7.1 μmol/mg of protein, respectively (*p*-value _Student_ < 0.0001). Concentrations of some sphingomyelins and only a fraction of phosphatidylcholines were also decreased in IUGR placentas compared to control placentas. However, the proportion of unsaturated fatty acyls, including monounsaturated (MUFA) and polyunsaturated (PUFA) acyl moieties of diacyl phosphatidylcholines (PC aa), was significantly increased in IUGR placentas compared to controls (median fold change of 1.47, *p*-value _Wilcoxon_ = 0.0002).

### 3.3. Metabolomic Signature of P2 Cohort

**Figure 2:**
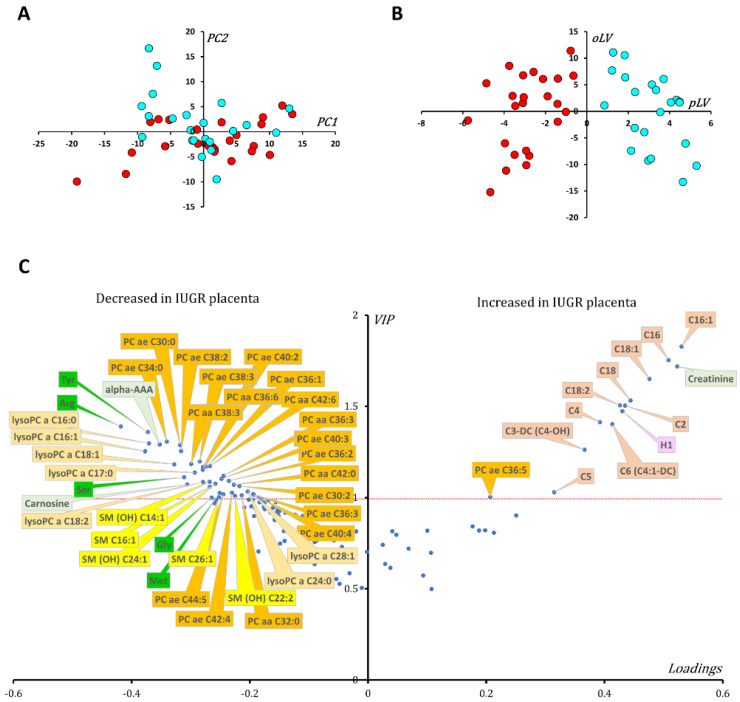
Multivariate statistical analysis of the metabolomic data from P2 cohort samples. IUGR (red dots) and control (blue dots) do not group together in the scatter plot of the PCA first principal plan (**A**), nor do any outliers appear according to Hotelling’s T2. However, projecting samples onto the predictive latent variable (pLV) in the OPLS-DA model enables good discrimination between IUGR and control samples (**B**). Loadings vs. VIPs (volcano plot, (**C**)) show increased concentrations of short (C2, C4, C4-OH and C5) and long (C16, C16:1, C18, C18:1, C18:2) chain acylcarnitine species along with creatinine, the sum of hexose (H1) and one acyl-alkyl phosphatidylcholine (PC ae 36:5). On the other hand, many lipidic metabolites, including lysophosphatidylcholine (lysoPCs), sphingomyelin (SM) and hydroxy sphingomyelin (SM(OH)) as well as phosphatidylcholine (PC) species are decreased. The concentration of some polar metabolites is also decreased in IUGR placentas, such as the amino acids tyrosine (Tyr), arginine (Arg), glycine (Gly), serine (Ser) and methionine (Met), as well as biogenic amines, including alpha-aminoadipic acid (alpha-AAA) and carnosine. For phosphatidylcholines, the sum of the length of the two acyl or acyl-alkyl groups is noted after the C and is followed by the number of double bonds. The same notation is used to represent the length and the number of double bonds in the acyl chain of sphingomyelins and lysophosphatidylcholine species. Color code: amino acids: green; acylcarnitine species: brown; biogenic amines: light green; lysophosphatidylcholine species: light orange; phosphatidylcholine species: dark orange; sphingomyelins and hydroxy sphingomyelins: yellow; sum of hexose or H1: pink.

From 188 measured metabolites in the kit, 139 were retained for the statistical analysis (see Supplementary Table S2). Principal component analysis (PCA) showed no outliers nor spontaneous sample grouping (Figure 2A). As for the P1 cohort, OPLS-DA provided good discrimination between groups (R^2^Y = 0.76) as observed in Figure 2B, with good predictive capabilities and with a low tendency towards overfitting (Q^2^Y cum = 0.51; *p*-value _CV-ANOVA_ < 0.003; Q^2^Y cum-perm = −0.64). Metabolite ranking according to VIP and loadings can be visualized in Figure 2C.

**Figure 3:**
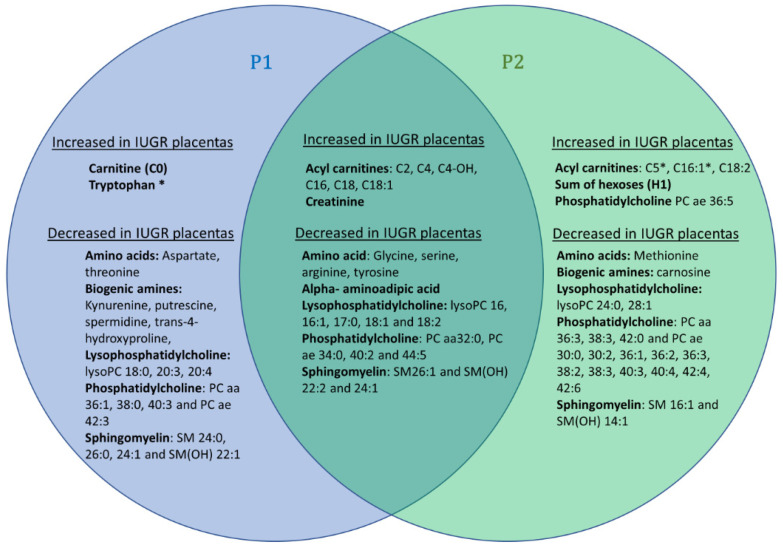
Venn diagram showing common discriminant metabolites in placenta P1 and/or P2 cohorts. Discriminant P1 metabolites have been drawn in a blue circle whilst important P2 metabolites have been included in a green circle. The intersection of both signatures comprises 25 metabolites, all varying in the same way in both cohorts: increased levels of 6 acyl-carnitines and creatinine and decreased levels of glycine, serine, arginine, tyrosine, alpha-aminoadipic acid, five lysophosphatidylcholine species (lysoPC), one diacyl phosphatidylcholine (PC aa 32:0) and three alkyl-acyl phosphatidylcholine (PC ae) species and 4 (hydroxy)sphingomyelin species (SM (OH) and SM, respectively). For the acylcarnitine, lysophosphatidylcholine, phosphatidylcholine and (hydroxy)sphingomyelin species, the sum of the length of the one or two acyl or acyl-alkyl groups is noted after “C”, “lysoPC”, “PC” and “SM(OH)” or “SM”, respectively, and is followed by the number of double bonds. * Indicates metabolites measured in only one cohort because they were out of range in the other cohort.

This supervised multivariate model uncovers 25 metabolites commonly modified in P1, all varying in the same direction, as shown in the Venn diagram presented in Figure 3). This replication of our study on two distinct cohorts reinforces the reliability of the results commonly obtained in both cohorts, but it should be noted that the disparities observed between these two cohorts may also be partly due to the greater prematurity and severity of the growth delay observed in the IUGR group of the P2 cohort. Indeed, the P2 cohort has slightly more discriminating total metabolites (*n* = 51), notably phosphatidylcholine species, than the P2 cohort (*n* = 44).

It should be noted that the values for tryptophan were below the lower limit of quantification for this P2 cohort and thus were not taken into consideration in the statistical analysis.

Similar to the P1 cohort, the ratio between acetyl and propionyl carnitines (C2 + C3) and free carnitine (C0) was significantly increased in P2 IUGR placentas (median fold change of 1.4, *p*-value _Wilcoxon_ = 0.017). Additionally, the ratio of unsaturated to saturated fatty acids was higher in IUGR placentas compared to controls (median fold change of 1.2, *p*-value _Wilcoxon_ = 0.0029).

### 3.4. Cord Blood Metabolomics

**Figure 4:**
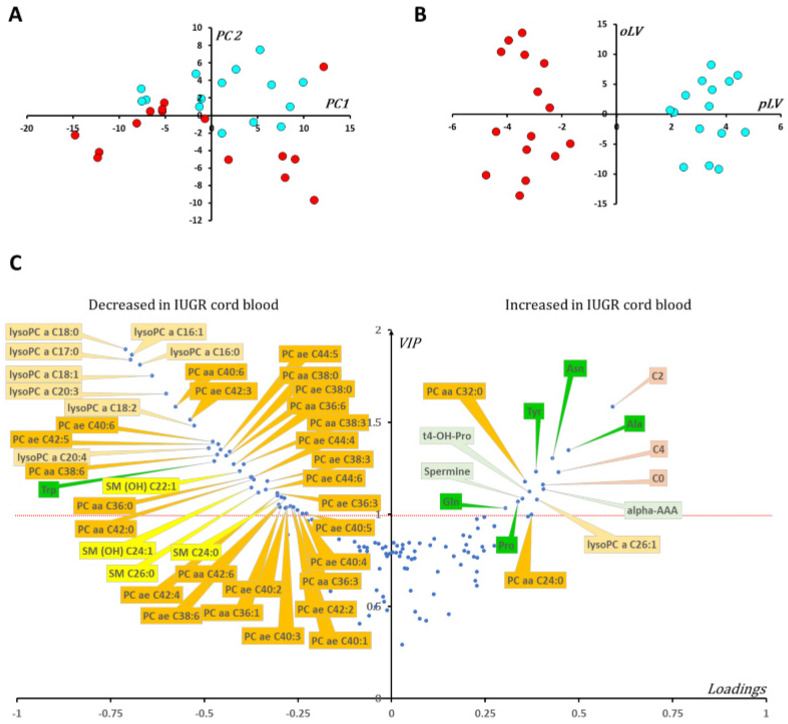
Multivariate statistical analysis of the metabolomic data from cord blood samples. (**A**) IUGR newborns (red dots) tend to have negative values in the second principal component (PC2) whilst control newborns (blue dots) have positive values in this component. There was no outlier according to Hotelling’s T2 distance. (**B**) The predictive latent variable (pLV) correctly predicts samples allocation according to IUGR status. Loadings vs. VIPs (volcano plot, (**C**)) show increased concentrations of carnitine (C0) and two short chain acylcarnitine species (acetyl (C2) and butyryl (C4) carnitine, respectively) along with five amino acids (alanine (Ala), asparagine (Asn), tyrosine (Tyr), glutamine (Gln) and proline (Pro)), three biogenic amines (alpha-aminoadipic acid (alpha-AAA), trans-4-hydroxyproline (t4-OH-Pro) and spermine), one lysophosphatidylcholine (lysoPC) and two diacyl phosphatidylcholine (PC aa) species. On the other hand, many lipid species including diacyl- and alkyl-acyl-phosphatidylcholines, sphingomyelins and lysophosphatidylcholines of less than 22 carbons are found diminished in the cord blood plasma of newborns diagnosed IUGR compared to controls. The amino acid tryptophan is also relatively decreased in the IUGR cohort compared to controls. For phosphatidylcholines, the sum of the length of the two acyl or acyl-alkyl groups is noted after the C and is followed by the number of double bonds. The same notation was used to represent the length and the number of double bonds in the acyl chain of sphingomyelins and lysophosphatidylcholine species. Color code: amino acids: green; acylcarnitine species: brown; biogenic amines: light green; lysophosphatidylcholine species: light orange; phosphatidylcholine species: dark orange; sphingomyelins and hydroxy sphingomyelins: yellow.

One hundred and forty-one measured metabolites were retained for the statistical analysis of the plasma of cord blood (see Supplementary Table S3). Principal component analysis (PCA) showed no outliers, but a trend towards group distinction was observed in control and IUGR samples, which had positive and negative values, respectively, in the second principal component PC2 (Figure 4A). OPLS-DA analysis enabled high group discrimination (R^2^Y = 0.95) as observed in Figure 4B, with good predictive capabilities and low overfitting (Q^2^Y cum = 0.72; *p*-value _CV-ANOVA_ < 0.0002; Q^2^Y cum-perm = −0.61). Metabolite ranking according to VIP and loadings are displayed in Figure 4C.

Figure 4C shows a deep lipid remodeling in the blood of IUGR newborns with decreased levels of lysophosphatidylcholines with acyl chain of less than 22 carbon atoms, many phosphatidylcholine species, and some sphingomyelins. The ratio of lysophosphatidylcholines to phosphatidylcholines, measuring phospholipase activity, was significantly diminished in IUGR newborns (median fold change of 0.65, *p*-value _Wilcoxon_ < 0.0001). Contrary to what was observed in placenta cohorts, the ratio of unsaturated to saturated fatty acids moieties in phosphatidylcholine molecules was significantly diminished in the plasma of IUGR newborns (median fold change of 0.84, *p*-value _Wilcoxon_ = 0.0043). The same inversed situation was also observed for some polar metabolites. Indeed, tyrosine and alpha-aminoadipic acid concentrations were found to be relatively decreased and tryptophan relatively increased in the plasma samples of IUGR newborns compared to controls. The plasma’s metabolomic signature was also characterized by elevated concentration of carnitine (C0), acetyl(C2) and butyryl(C4) carnitine species as well as the amino acids alanine, asparagine, proline, and glutamine. Plasmatic concentrations of biogenic amines trans-4-hydroxyproline and the polyamine spermine were also elevated in IUGR samples.

## 4. Discussion

The replication in two cohorts of IUGR and control placentas, recovered from women who underwent vaginal and cesarean delivery, reveals a set of 25 discriminating metabolites, similarly modified in both cohorts. In the case of IUGR, six short- and medium-chain AC and creatinine show increased concentrations, whereas four amino acids (glycine, serine, arginine, and tyrosine), alpha-aminoadipate, and twelve glycerophospholipids (five lysophosphatidylcholines, four phosphatidylcholine and three sphingomyelins) show decreased concentrations. Such a decrease in glycerophospholipid concentrations is also found the IUGR blood cords compared to controls (seven lysophosphatidylcholines, 27 phosphatidylcholines and four sphingomyelins, yet only two increased phosphatidylcholines and one lysophosphatidylcholine). Only two short-chain acylcarnitine species are increased, as in the placentas, in cord blood from IUGR compared to controls, with the addition of free carnitine (C0), which is also increased. In contrast to placentas, in umbilical cord blood plasma, creatinine does not appear to be discriminating and alpha-aminoadipate is found to be increased. Lastly, with respect to the amino acids, only tyrosine appears to be discriminating in cord blood as it is in placentas, but inversely, with an increased concentration in cord blood. Four other amino acids not present in the placental signature appear to be increased in blood (alanine, asparagine, glutamine, and proline), and tryptophan appears to be decreased.

Creatinine is a degradation product of creatine phosphate that plays an important role in energy homeostasis through the ability of creatine-phosphate to phosphorylate ADP. The blood level of creatinine depends mainly on the production of creatine by skeletal muscle and its elimination by kidneys. Increased concentrations of creatinine have already been reported in the urine of IUGR newborns [7], in cord blood of IUGR patients [11] and in fetal umbilical venous plasma of growth-restricted fetal pigs [16], while the concentration of its precursor creatine has been shown to be increased in the umbilical cord blood plasma of patients with IUGR compared to controls [12]. Here, we show for the first time, to our knowledge, that creatinine has also elevated levels in the placenta of IUGR patients. As the placenta is known for its ability to perform creatine biosynthesis [17], the increase of this metabolite could result from mitochondrial dysfunction due to hypoxia, as has been suggested in the case of preeclampsia, where a similar elevation of creatine concentration can be seen [18].

The fetus is an “essentially glycolytic organism” and the paramount importance of placental-to-fetal glucose transfer is a well-accepted paradigm. Interestingly, hexoses, which are the other main energy source for mitochondria in the placental syncytium (PS), were also found to be increased in the most severe IUGR P2 cohort (Figure 3 and Figure 4). The PS is equipped with the whole machinery for fatty acid oxidation and Shekhawat et al. have demonstrated that fatty acid oxidation in PS was comparable to or even greater than that in cultured human fibroblasts [19]. In our study, we observed a larger carnitine pool (free carnitine plus AC) in IUGR placentas in both P1 and P2 cohorts compared to controls (*p*-value _P1_,_Student test_ = 0.019 and *p*-value _P2_,_Student test_ < 0.001, respectively) accompanied by significant alteration of AC/C0 ratios (*p*-value _P1_,_Wilcoxon test_ = 0.005 and *p*-values _P2_,_Wilcoxon test_ = 0.006, respectively). These results point toward increased carnitine accumulation and fatty acid oxidation in IUGR PS mitochondria. However, in the relatively hypoxic environment associated with IUGR, fatty acid oxidation is probably incomplete, resulting in further AC accumulation. Such acylcarnitine species and fatty acid accumulation has also been reported in umbilical cord blood [20] and in the blood of IUGR newborns [21]. Additionally, a negative correlation between birthweight and acylcarnitine species concentration in blood was recently identified in larger cohorts [22]. According to these authors, this metabolic signature could reflect insulin resistance that is closely related to mitochondrial energy metabolism. In this configuration of insulin resistance, fatty acid oxidation would be an important source of energy for the PS.

Increased α-aminoadipic acid in newborn blood could be another effect of a state of insulin resistance. Indeed, α-aminoadipic acid, a metabolite of lysine catabolism, has been identified as an early biomarker of insulin resistance [23,24]. Interestingly, concentrations of α-aminoadipic acid were lower in IUGR placentas compared to control placentas in both cohorts. Taking together both placental and newborn blood data, the hypothesis of diminished placental clearance of fetal α-aminoadipic acid in IUGR pregnancies seems plausible. In pregnant sheep, Wilkes et al. provided evidence of the placenta’s important role in clearing fetal α-aminoadipic acid after a maternal lysine load [25]. To our knowledge, no study has been carried out in humans to investigate the role of the placenta in eliminating fetal blood α-aminoadipic acid.

Concerning amino acids, whose altered concentrations are generally attributed to altered transport in the IUGR deficient placenta [16], our signatures observed in placenta and cord blood diverge considerably. Tyrosine appears discriminating in the two samples, but in opposite direction. Others amino acids (glycine, serine, and arginine) are decreased in placenta, while five others, not present in the placental signature, appear to be either increased (alanine, asparagine, glutamine and proline) or decreased (tryptophan) in blood. The discriminant amino acids found by Bahado-Singh et al. [13] in IUGR placenta are not quite the same as ours, but the decreased concentration of tyrosine and glycine in both studies reinforces their pathophysiological importance. Similarly, in IUGR cord blood, according to the different studies, changes in the concentrations of tyrosine, alanine, glutamine, serine, proline, and tryptophan have been reported, highlighting their pathophysiological importance [4,12,16,21]. Creatinine, a surrogate of creatine synthesis, was significantly increased in IUGR placentas. Creatine is synthetized from glycine and arginine. Interestingly, both glycine and arginine were decreased in P1 and P2 IUGR samples. It is tempting to speculate about enhanced local creatine synthesis in IUGR placentas aiming to improve spatial energy allocation through phosphocreatine in this tissue.

Our signature also shows a sharp rearrangement of glycerophospholipids in IUGR, with a massive decrease in their concentration in P1 and P2 cohorts and in cord blood. A global decrease in phosphatidylcholines has already been reported in the placenta of patients with fetal growth restriction [13] as well as in the blood of IUGR patients and in the blood of a rat model of IUGR [26]. In IUGR placentas the ratio of unsaturated to saturated fatty acids moieties in phosphatidylcholine species was significantly higher compared to controls. The opposite was observed in cord blood samples, illustrating a complex interplay of metabolic changes between the fetal and the placental compartments. Interestingly, the concentration of lysophosphatidylcholines in cord blood has been shown to positively correlate with birth weight [9]. The ratio of lysophosphatidylcholines to phosphatidylcholines, measuring phospholipase A2 activity, was significantly diminished in IUGR cord blood samples, but not at the placental level, showing, once again, the complexity of metabolic interactions inside the fetoplacental unit. This remodeling of glycerophospholipids could be either due to a structural modification of the placenta, with the phospholipids being the most important components of biological membranes, or due to a more general modification of lipid metabolism relating to the energetic impairment. Indeed, a substantial disruption of lipid metabolism, including altered lipoprotein profiles has been shown in mother and fetuses with IUGR [27].

Our study is the second to explore the placenta of IUGR patients after that recently published by Bahado-Singh et al. [13]. It differs from that study in its use of a replicated placental cohort and in its comparison of the metabolomic profile of fetal umbilical cord blood. The disparity in pregnancy term between IUGR and control groups in the P2 cohort is a limitation of our study, since it is difficult to constitute a cohort of healthy patients with a similar term to IUGR. However, this disparity in pregnancy term is less marked in our P1 cohort. The fact that we obtained a common metabolomic signature in the two cohorts studied, despite this disparity of pregnancy terms, shows that the signature of the IUGR is predominant over that of the variations in the term of pregnancy. Our study confirms a deep remodeling of glycerophospholipids, already shown by Bahado-Singh et al., and the modifications of some amino acids. However, it also uncovered for the first time an altered saturated-to-unsaturated ratio of the acyl moieties forming these phospholipids and potentially diminished PLA2 activity in the plasma of IUGR newborns. It also reveals an increase in creatinine, alpha-aminoadipate and acylcarnitine species in the placenta obtained from IUGR pregnancies, pointing toward a disturbed energy metabolism with insulin resistance.

## 5. Conclusions

In summary, our study shows a profound alteration in the placenta of IUGR patients with respect to energy and lipid metabolism, with insulin resistance, increased activity of fatty acids oxidation, altered saturated-to-unsaturated fatty acids moieties in phosphatidylcholines and phospholipase activity. It is tempting to propose that this placental metabolic reprogramming could have a lasting effect on the fetus and that it may be responsible for the increased susceptibility to metabolic and cardiovascular diseases eventually observed in adulthood.

## Data Availability

All clinical and metabolomic data are presented in Supplementary Tables.

## Supplementary Materials

The following supporting information can be downloaded at: https://www.mdpi.com/article/10.3390/biomedicines10061411/s1, Table S1: Metabolomic raw data of the P1 cohort. Table S2: Metabolomic raw data of the P2 cohort. Table S3: Metabolomic raw data of the cord blood cohort.
