# Supplementary material for: A Metabolomic Profiling of Intra-Uterine Growth Restriction in Placenta and Cord Blood Points to an Impairment of Lipid and Energetic Metabolism
Source: Biomedicines. 2022 Jun 15;10(6):1411. doi: 10.3390/biomedicines10061411 (PMC9220006; doi:10.3390/biomedicines10061411)
Supplement: Supplementary file 1 [file biomedicines-10-01411-s001.zip › biomedicines-1735489-supplementary.pdf]

A metabolomic profiling of intra-uterine growth restriction in placenta and cord blood points to an impairment of lipid and energetic metabolism

Supplementary Tables S1–S3

Supplementary Table S1. Description of the placenta cohort P1 (N = 40) and comparison of characteristics of the IUGR and control groups. Quantitative data are expressed as an average and quartiles as the standard deviation. Qualitative data are expressed in numbers and percentages N (%). Significantly different data are presented in bold (p <0.05). BMI = body mass index, IUGR = intrauterine growth restriction.

			**Control Group n = 20**	**IUGR Group n = 20**	**p**
**Maternal data**	**Age (years)**		31.9 (5.2)	28.7 (4.9)	0.05
	**BMI**		24.1 (5.4)	23.8 (3.7)	NS
	**Tobacco during pregnancy**		3 (15)	8 (40)	0.06
**Obstetric data**	**Gestity**		3.6 (1.9)	2.7 (2.1)	NS
	**Parity**		2.3 (1.3)	1.4 (1.0)	**0.02**
	**Type of delivery**	**Vaginal delivery**	0 (0)	5 (25)	**0.005**
		**C-section**	20 (100)	15 (75)	
	**IUGR**		0 (0)	20 (100)	**<0.001**
**Newborn data**	**Gestational age**		38.7 (0.6)	36.4 (1.9)	**<0.001**
	**Gender**	**Boy**	9 (45)	9 (45)	NS
		**Girl**	11 (55)	11 (55)	
	**Birth weight (z-score)**		-0.1 (0,8)	-2.2 (0.6)	**<0.001**
	**Birth weight (g)**		3289.3 (400.3)	1817.7 (392.1)	**<0.001**
	**Birth size (z-score)**		-0.4 (0.8)	-2.3 (0.8)	**<0.001**
	**Birth size (cm)**		49.0 (1.6)	42.4 (2.1)	**<0.001**
	**Birth head circumference (z-score)**		-0.1 (0.9)	-1.4 (0.8)	**<0.001**
	**Birth head circumference (cm)**		34.3 (1.4)	30.7 (2.0)	**<0.001**
	**APGAR**		10.0 (0.2)	9.4 (2.3)	NS
	**Resuscitation at birth**		0 (0)	2 (10)	NS
	**Hospitalization**		0 (0)	15 (75)	**<0.001**

Supplementary Table S2. Description of the placenta cohort P2 (n = 46) and comparison of characteristics of the IUGR and control groups. Quantitative data are expressed as an average and quartiles as the standard deviation. Qualitative data are expressed in numbers and percentages N (%). Significantly different data are presented in bold (p <0.05). BMI = body mass index, IUGR = intrauterine growth restriction.

			**Control Group n = 22**	**IUGR Group n = 24**	**p**
**Maternal data**	**Age (years)**		32.0 (4.1)	29.9 (6.5)	NS
	**BMI**		27.5 (6.9)	26.3 (9.3)	NS
	**Tobacco during pregnancy**		1 (4.5)	6 (25)	0.07
**Obstetric data**	**Gestity**		3.0 (1.9)	2.4 (2.0)	NS
	**Parity**		2.1 (1.1)	1.5 (1.4)	NS
	**Type of delivery**	**Vaginal delivery**	0 (0)	2 (8)	NS
		**C-section**	22 (100)	22 (92)	
	**IUGR**		0 (0)	24 (100)	**<0.001**
**Newborn data**	**Gestational age**		39.0 (0.7)	31.3 (3.4)	**<0.001**
	**Gender**	**Boy**	11 (50)	10 (45)	NS
		**Girl**	11 (50)	14 (55)	
	**Birth weight (z-score)**		-0.1 (0.8)	-1.8 (0.7)	**<0.001**
	**Birth weight (g)**		3144.1 (392.0)	1155.0 (615.7)	**<0.001**
	**Birth size (z-score)**		-0.5 (0.9)	-2.2 (1.1)	**<0.001**
	**Birth size (cm)**		48.9 (2.2)	36.7 (5.2)	**<0.001**
	**Birth head circumference (z-score)**		-0.1 (0.9)	-1.2 (1.0)	**<0.001**
	**Birth head circumference (cm)**		34.5 (1.5)	27.1 (3.5)	**<0.001**
	**APGAR**		10 (0)	9.2 (1.2)	**0.003**
	**Resuscitation at birth**		0 (0)	17 (85)	**<0,001**
	**Hospitalization**		0 (0)	22 (92)	**<0.001**

Supplementary Table S3. Description of the cord blood cohort (n = 30) and comparison of characteristics of the IUGR and control groups. Quantitative data are expressed as an average and quartiles as the standard deviation. Qualitative data are expressed in numbers and percentages N (%). Significantly different data are presented in bold (p < 0.05). BMI = body mass index, IUGR = intrauterine growth restriction.

			Control Group n = 15	IUGR Group n = 15	p
**Maternal data**	**Age (years)**		33.1 (4.2)	26.9 (5.4)	**0.002**
	**BMI**		25.7 (7.7)	25.5 (4.7)	NS
	**Tobacco during pregnancy**		0 (0)	4 (27)	0.09
**Obstetric data**	**Gestity**		3.1 (2.1)	2.8 (2.3)	NS
	**Parity**		2.1 (1.0)	1.5 (1.1)	NS
	**Type of delivery**	**Vaginal delivery**	0 (0)	4 (27)	0.07
		**C-section**	15 (100)	11 (73)	
	**IUGR**		0 (0)	15 (100)	**<0.001**
**New-born data**	**Gestational age**		39.1 (0.7)	35.2 (2.8)	**<0.001**
	**Gender**	**Boy**	5 (33)	7 (47)	NS
		**Girl**	10 (67)	8 (53)	
	**Birth weight (Zscore)**		0.0 (0.8)	-1.8 (0.7)	**<0.001**
	**Birth weight (g)**		3400.0 (444.5)	1760.5 (560.6)	**<0.001**
	**Birth size (Zscore)**		-0.4 (1.2)	-2.1 (0.8)	**<0.001**
	**Birth size (cm)**		49.4 (2.8)	41.3 (3.3)	**<0.001**
	**Birth head circumference (Zscore)**		-0,3 (1,1)	-1.3 (0.7)	**0,01**
	**Birth head circumference (cm)**		34.0 (1.9)	30.0 (2.6)	**<0.001**
	**APGAR**		9.9 (0.3)	9.8 (0.8)	NS
	**Resuscitation at birth**		0 (0)	2 (13)	NS
	**Hospitalization**		0 (0)	12 (80)	**<0.001**
